# From Footprints to Forecast: Baropodometry for Fall Risk Identification and Mobility Classification Among Pilgrims

**DOI:** 10.3390/jcm15051970

**Published:** 2026-03-04

**Authors:** Hanan A. Demyati, Abdulelah M. Radhwan, Yasir A. Alrubaiani, Raneem Y. Alshahrani, Mashael H. Allabban, Mohammed O. Aloufi, Yousef H. Aljabri, Layla M. Abdullrhman, Ali M. Albarrati

**Affiliations:** 1 Rehabilitation Department, Prince Sultan Armed Forces Hospital, Madinah Al-Munawwarah 42317, Saudi Arabia; 2 Rehabilitation Sciences Department, College of Applied Medical Sciences, King Saud University, Riyadh 11451, Saudi Arabia; 3 School of Healthcare Sciences, Cardiff University, University Hospital of Wales, Cardiff CF14 4XN, UK

**Keywords:** fall risk assessment, mobility screening, pilgrims

## Abstract

**Background/Objectives**: Hajj is a major annual mass gathering. It requires prolonged walking under conditions of fatigue, heat stress, and crowd density, which increases mobility difficulties and fall risk, particularly among older adults and individuals with chronic diseases. Therefore, rapid operational mobility screening is required to identify risk and plan mobility. To support an operational mobility-classification workflow in a pre-Hajj setting, this study evaluated whether Timed Up and Go (TUG)-based stratification, combined with spatiotemporal gait and plantar pressure measurements, differentiates fall-risk categories. **Methods**: We conducted a cross-sectional study at a seasonal medical center near Al-Haram in Madinah Al-Munawwarah (21 May–3 June 2025) within the “I Lean On It” screening initiative. Participants completed the TUG and dynamic baropodometric gait assessments. We stratified the risk of falling as low (≤10 s), moderate (10.1–13.5 s), and high (>13.5 s) according to the TUG performance. We performed between-group comparisons using the Kruskal–Wallis test and evaluated the associations using Spearman’s correlation analysis. **Results**: Participants were classified as having low (*n* = 103), moderate (*n* = 24), or high (*n* = 29) fall risk. TUG performance significantly increased across the fall-risk groups. Significant between-group differences were observed in cadence, half-step length, walking speed, test duration, and functional mobility, whereas plantar pressure magnitude and gait symmetry did not differ significantly. Spearman correlation analysis showed significant negative correlations between TUG time and sex (rs = −0.357), half-step length (rs = −0.617), walking speed (rs = −0.577), and cadence (rs = −0.420). Significant positive correlations were observed with weight-bearing time (right: rs = 0.584; left: rs = 0.461), test duration (rs = 0.376), and number of steps acquired (rs = 0.356) (all *p* ≤ 0.003). Overall, TUG performance was primarily associated with dynamic gait and functional mobility. **Conclusions**: Integrated functional mobility and spatiotemporal gait screening significantly differentiate fall risk and provide clinically actionable mobility-support guidance in a mass-gathering pre-Hajj clinical workflow.

## 1. Introduction

Hajj is one of the world’s largest recurring mass gatherings, attracting more than two million pilgrims to Makkah, Saudi Arabia, each year and posing substantial public-health challenges, particularly in relation to mobility, endurance, and fall prevention. Pilgrims are required to walk unpredictable and often prolonged distances over several days. They frequently experience crowd congestion, restricted personal space, variable pacing, heat stress, and cumulative physical fatigue. These conditions, including prolonged walking and environmental stressors, substantially increase the risk of musculoskeletal strain and falls, especially among older adults and individuals with pre-existing mobility limitations or chronic diseases [1,2]. Falls during Hajj have been consistently associated with advanced age, fatigue, inappropriate footwear, and high crowd density, and represent a major cause of injury requiring medical attention during the pilgrimage season [3,4,5]. To address these challenges, effective mass-gathering health management relies on rapid, standardized, and actionable decision-making.

Contemporary mass-gathering medicine frameworks emphasize the early identification of vulnerable individuals and the translation of risk information into operational actions, such as prioritization, routing, staffing, and assistive-device allocation [6,7]. Effective mobility screening generates clear and interpretable categories that support immediate decisions. Continuous test values may be difficult to operationalize in crowded, time-limited environments, making categorized results more effective in such settings.

Several validated instruments are commonly used to assess functional mobility and fall risk, including the Short Physical Performance Battery (SPPB), gait speed tests over short distances (e.g., 4 m), longer walk tests (e.g., 400 m), and the Timed Up and Go (TUG) test. These assessments capture complementary components of physical function, such as balance, lower extremity strength, endurance, and gait performance [8]. The TUG is one of the most widely used tools for quantifying functional mobility and screening fall risk. It evaluates a sequence of movements, including standing from a seated position, walking, turning, returning, and sitting, which closely reflect real-world ambulation demands [9]. TUG performance shows moderate correlations with longer walking tests and functional capacity, supporting its relevance as a proxy for overall mobility [10,11]. A threshold of approximately ≥13.5 s is commonly used to indicate an elevated fall risk, although optimal cut-off values vary across populations and clinical contexts [12,13]. Owing to its rapid administration, low cost, and ease of interpretation, the TUG is especially suited to mass-gathering settings, where standardized and objective screening must be completed efficiently.

While the TUG test is widely used for global functional mobility assessment, a significant gap exists in understanding how specific aspects of gait control are compromised, particularly under the strenuous conditions of mass gatherings like Hajj. This lack of detailed insight limits the ability to develop truly comprehensive and operational fall-risk stratification frameworks [14,15,16,17]. These observations support the integration of objective gait quality markers alongside functional tests to more accurately represent the real-world fall risk during sustained ambulation.

Baropodometric and pressure platform systems provide quantitative measures of plantar pressure distribution, weight transfer patterns, and center-of-pressure behavior during walking. Cross-sectional studies have demonstrated differences in dynamic plantar-pressure parameters between individuals at higher versus lower risk of falls, supporting their value as biomechanical indicators of gait instability [18,19]. Functional mobility tests, spatiotemporal gait analysis, and plantar pressure measurements each offer individual merits. However, a critical research gap exists in their integrated application within a structured, operational framework. This gap is particularly evident for rapid, actionable risk stratification in mass-gathering contexts, where isolated measurements are often insufficient.

In addition to risk identification, mobility screening can provide targeted mobility support. Evidence indicates that appropriate prescription of walking aids can modify spatiotemporal gait parameters, improve balance control, reduce lower limb loading, and enhance endurance during prolonged walking, especially in older adults exposed to physically demanding environments [20,21,22]. Clinical guidelines emphasize that walking aids should be prescribed and reviewed based on functional walking performance, safety considerations, and environmental demands rather than diagnosis alone [23]. Finally, translating assessment findings into operational actions requires effective communication. Adapting simplified color-coded classification systems (e.g., green–yellow–red) is consistent with the established principles of rapid visual communication, particularly in crowded environments where verbal handovers may be unreliable. Color-based categorization has long been used in mass-casualty and emergency triage to convey priority and resource needs efficiently, whereas identification tools such as cards and barcode-enabled tags have been shown to improve coordination and information transfer during disaster responses [24,25].

To address this critical research gap, the lack of an integrated, operational mobility risk stratification framework for mass gatherings, this study integrated functional mobility performance, spatiotemporal gait characteristics, and plantar pressure measurements within a structured operational framework. Our aim was to demonstrate how such an approach, tailored to pilgrimage settings, can effectively classify mobility, inform assistive device recommendations, and identify fall risk and walking difficulties during prolonged ambulation in these unique environments.

## 2. Methods

### 2.1. Study Design and Participants

This cross-sectional observational study employed convenience sampling and was conducted at a seasonal medical center near Al-Haram in Madinah Al-Munawwarah, Saudi Arabia, before the Hajj season. Data collection occurred between 21 May and 3 June 2025, during clinic operational hours (5:00 PM–10:00 PM), corresponding to the peak attendance for pre-Hajj medical assessment. The study was embedded within the “I Lean On It” mobility screening initiative, designed to support rapid functional triage in mass-gathering healthcare settings.

All participants who presented to the clinic during operational hours were eligible for screening. Initial visual mobility triage was performed at the clinic entrance, consistent with established emergency and mass-gathering practices aimed at rapidly identifying functional mobility limitations [26,27]. Individuals were referred for physiotherapy assessment if they demonstrated (1) visibly prolonged walking time (>10 s over an approximate 6 m distance), (2) observable gait, balance, or postural instability, or (3) current wheelchair use or dependency. We excluded participants only if they were wheelchair users and unable to get up and perform the physical performance tests. This approach was intentionally adopted to reflect the real-world mobility characteristics of pilgrims with heterogeneous health profiles, consistent with the pragmatic objectives of mass-gathering screening [1,6].

All assessments were conducted by licensed physiotherapists who were independent of the study design and statistical analyses. The assessors were blinded to the fall-risk classification during data collection to reduce observer bias. Each participant completed two walking trials, and the average value was used for the analysis to minimize intra-individual variability and enhance measurement reliability [28].

### 2.2. Fall-Risk Assessment and TUG Classification

Fall risk was evaluated using the TUG test, a widely validated functional mobility assessment involving standing from a seated position, walking 3 m, turning, returning, and sitting [9]. The TUG demonstrates strong inter-rater and test–retest reliability (intraclass correlation coefficients 0.81–0.91) in older adults and clinical populations [29].

To enhance clinical interpretability and avoid ambiguity associated with single cutoff thresholds, a three-tier TUG-based fall-risk stratification framework was applied.

- Low fall risk: TUG ≤ 10 s.
- Moderate fall risk: TUG 10.1–13.5 s.
- High fall risk: TUG > 13.5 s.

These thresholds are consistent with the established literature and reflect gradations in functional mobility relevant to the prolonged walking demands encountered during pilgrimage activities [12,13].

### 2.3. Assistive-Device Trial and Post-Assessment TUG

Participants who were classified as having a moderate or high fall risk underwent a standardized assistive device trial using a walking cane. We used plantar pressure analysis to identify the side with greater load bearing, and a cane was prescribed for the contralateral side. Following standardized fitting and brief instructions, the TUG test was immediately repeated to assess the functional response to cane use.

This post-assessment TUG was not intended as an intervention outcome but served as a clinical verification step to support the operational mobility classification. Post-cane TUG values were used exclusively for mobility card assignment and were excluded from the primary inferential statistical analyses.

### 2.4. Mobility Classification System and Color-Coded Cards

A simplified color-coded mobility identification system (green, yellow, and red) was implemented as an applied extension of the assessment process to support real-time decision-making and communication in mass-gathering healthcare logistics. The classification was based on TUG performance and response to assistive device testing.

- Green card: Assigned to participants with a low fall risk or to those with moderate or high risk who demonstrated functional improvement following cane use, indicating independent ambulation with or without a cane.
- Yellow card: Assigned to participants with a high fall risk who showed no improvement or deterioration in post-cane TUG performance, indicating the need for a cane for short distances and wheelchair support for longer distances.
- Red card: Assigned to individuals with permanent wheelchair dependency identified during triage.

Each card incorporated a barcode-linked system that provided individualized mobility guidance in Arabic and English, facilitating coordinated care across healthcare, accommodation, and transportation services. Card allocation was not analyzed as an outcome variable but represented a key translational component of the screening framework [26].

### 2.5. Anthropometric Measures

Height and weight were measured using standardized procedures, and the body mass index (BMI) was calculated accordingly. BMI was included because of its established association with altered gait mechanics, increased plantar pressure, reduced step length, and elevated fall risk, particularly under prolonged walking and fatigue-inducing conditions [30].

### 2.6. Gait and Plantar-Pressure Parameters

Static and dynamic gait parameters were collected using a FreeMed baropodometric platform with FreeStep software rev 2.0 (Sensor Medica Srl, Rome, Italy). This system provides high-resolution plantar pressure mapping and spatiotemporal gait metrics [31]. The variables analyzed included cadence, step length, walking speed, weight-bearing time, plantar-pressure distribution, foot progression angle, and symmetry index [31]. Baropodometric pressure platforms and in-shoe pressure systems have demonstrated good-to-excellent test–retest reliability and strong agreement with established gait analysis methods [32].

These systems are considered valid tools for the quantitative assessment of foot loading patterns, gait symmetry, and temporal characteristics of gait [19].

### 2.7. Statistical Analysis

Statistical analyses were performed using IBM SPSS Statistics Version 26.0 (IBM Corp., Armonk, NY, USA). Descriptive statistics were used to summarize the demographic, clinical, and gait characteristics. The Shapiro–Wilk test for normality revealed a significant deviation from the normal distribution for the majority of continuous variables (*p* < 0.05); consequently, non-parametric methods were utilized. Descriptive data are presented as median and interquartile range (IQR) where appropriate. Group comparisons across the low-, moderate-, and high-risk categories were conducted using the Kruskal–Wallis H test. Associations between TUG performance, spatiotemporal gait parameters, plantar pressure measurements, and fall risk classification were examined using Spearman’s rank-order correlation coefficients. Statistical significance was set at *p* < 0.05 [33].

## 3. Results

### 3.1. Participant Characteristics and Mobility Classification

A total of 180 individuals were screened during the study period. Of these, 24 were excluded because they were unable to perform physical performance tests. The physical and clinical characteristics of the participants are presented in Table 1. Approximately half of the participants reported at least one chronic medical condition, most commonly hypertension or diabetes mellitus. Musculoskeletal complaints were reported by 59.8% of participants, predominantly nonspecific pain and low back pain (LBP). Participants were classified as having low (*n* = 103), moderate (*n* = 24), or high (*n* = 29) fall risk based on their TUG test performance (Table 1). An increased fall risk was associated with older age and female predominance.

Participants aged ≥50 years comprised 58.3%, 79.2%, and 93.1% of the low-, moderate-, and high-risk groups, respectively. The prevalence of chronic diseases and musculoskeletal disorders increased progressively across the fall-risk categories. The need for assistive devices differed markedly between the groups. No cane was required among low-risk participants, whereas 91.7% of moderate-risk and 100% of high-risk participants required cane support during the assessment. Mobility card allocation demonstrated complete concordance with fall-risk classification: low-risk participants required no card, moderate-risk participants received green cards, and high-risk participants received yellow cards.

### 3.2. Functional Mobility, Gait, and Plantar-Pressure Comparisons

Functional mobility and spatiotemporal gait parameters stratified by fall-risk category are presented in Table 2. Participants classified as having high fall risk demonstrated substantially poorer functional mobility and dynamic gait performance compared with those in the low- and moderate-risk groups. This pattern was reflected by progressively longer TUG times, lower cadence, shorter step lengths, slower walking speeds, and longer test durations across increasing fall-risk categories. Kruskal–Wallis testing demonstrated significant between-group differences in age, cadence, average half-step length, walking speed, test duration, and pre-assistive-device TUG performance (all *p* < 0.01). The pre-assistive-device TUG showed the strongest discriminatory capacity, demonstrating a very large effect size and robust differentiation between fall-risk categories based on functional mobility. The half-step length and walking speed also demonstrated large effect sizes, whereas cadence and test duration showed moderate effects. In contrast, BMI, symmetry index, and bilateral plantar pressure magnitudes did not differ significantly across the fall-risk groups and demonstrated negligible effect sizes. These findings indicate that global functional mobility and spatiotemporal gait parameters are more sensitive indicators of mobility-related fall risk than isolated plantar-pressure metrics when evaluated independently.

### 3.3. Association of TUG Performance with Spatiotemporal Gait and Functional Mobility Parameters

Spearman correlation analysis (Table 3) demonstrated significant negative correlations between TUG time and half-step length, walking speed, cadence, and sex, indicating higher TUG times among females and poorer functional performance with worsening gait characteristics. Significant positive correlations were observed between TUG time and weight-bearing time on both the right and left sides, as well as the test duration, number of steps acquired, and body mass index. No statistically significant correlations were found between the TUG time and age or symmetry index. Overall, TUG performance was more strongly associated with dynamic gait characteristics and functional mobility measures than with demographic characteristics or static loading symmetry.

## 4. Discussion

This study demonstrates that a structured, rapid mobility risk stratification framework integrating functional mobility testing, spatiotemporal gait analysis, and an operational classification system can meaningfully differentiate fall risk among pilgrims preparing for the Hajj. The findings support the central premise that actionable mobility screening, rather than isolated measurements, is essential in mass-gathering contexts where prolonged walking, cumulative fatigue, crowd density, and environmental stressors collectively amplify fall risk [1,2,6]. In such settings, translating biomechanical and functional assessments into operationally meaningful categories is particularly relevant for planning prevention and coordinating the services.

Our findings are consistent with the mass-gathering literature; a higher fall risk in this cohort was associated with older age and female predominance, as well as a greater burden of chronic disease and musculoskeletal complaints [3,4,5]. The progressive increase in participants aged ≥50 years across the low-, moderate-, and high-risk groups mirrors earlier reports identifying advanced age as a key determinant of falls during pilgrimage, likely reflecting age-related declines in balance control, muscle strength, gait adaptability, and fatigue tolerance [4,5]. Similarly, the higher representation of women in the moderate- and high-risk groups aligns with evidence suggesting sex-related differences in gait stability, pain prevalence, and fall vulnerability in older adults, particularly under physically demanding walking conditions [3,5,30].

A key finding of this study was the strong discriminatory capacity of the TUG-based stratification. Participants classified as high risk demonstrated markedly prolonged TUG times, with very large effect sizes, confirming the TUG test as a robust indicator of functional mobility limitation in this context [9,12,29]. Importantly, the use of three clinically meaningful TUG categories (≤10 s, 10.1–13.5 s, and >13.5 s) reduced the ambiguity inherent in single cutoff approaches and enabled clearer differentiation between moderate and high mobility risks. Individuals in these categories are likely to require different levels and types of mobility support rather than uniform interventions, particularly during the prolonged and crowded ambulation typical of the Hajj rituals [13]. Building on the TUG’s discriminatory capacity, this study identified specific spatiotemporal gait parameters that contribute to fall risk classification. Cadence, step length, walking speed, and test duration differed significantly across the risk groups and exhibited moderate-to-large effect sizes. These parameters reflect dynamic gait efficiency and neuromuscular control, both of which are essential for sustained walking in real-world conditions [15,16]. The reductions in step length and walking speed observed in high-risk participants are consistent with the cautious, compensatory gait strategies commonly reported in older adults and individuals at an increased risk of falling, particularly when balance confidence and reserve capacity are reduced [16].

In contrast, symmetry indices and plantar-pressure magnitudes did not independently distinguish fall-risk categories. While plantar-pressure assessment provides valuable biomechanical insight, these findings suggest that isolated pressure values may have limited standalone utility for fall-risk stratification in heterogeneous ambulatory populations [18,19]. Rather, plantar-pressure data appear more informative as supportive clinical measures, for example, in guiding footwear or assistive-device prescription than as primary discriminators of fall risk. This interpretation aligns with prior evidence indicating that spatiotemporal gait patterns and variability are more sensitive indicators of instability than absolute pressure metrics alone [18]. An important contribution of this study is the incorporation of assistive device verification into the screening process. Nearly all participants in the moderate- and high-risk groups required cane support, highlighting the high prevalence of previously unaddressed mobility needs prior to the structured assessment. The immediate post-cane TUG reassessment, although not intended as an intervention outcome, served as a pragmatic clinical verification step, allowing differentiation between individuals who could ambulate safely with minimal support and those who continued to demonstrate substantial functional limitations. This approach is consistent with best-practice recommendations emphasizing that walking aids should be prescribed based on functional performance, safety, and environmental demands rather than diagnosis alone [21,23].

The color-coded mobility classification system should be interpreted as an operational communication tool, rather than as evidence of clinical effectiveness. In this study, the system functioned as a structured mechanism to translate assessment findings into standardized mobility categories that could be readily recognized across healthcare, transportation, and support services. The concordance between fall-risk stratification and card allocation reflects the internal consistency of the framework, not a demonstrated impact on clinical outcomes. Nevertheless, the feasibility of linking objective mobility screening results to simple visual identifiers highlights a potential mechanism for improving coordination in mass-gathering environments, where rapid, non-verbal communication is often essential [1,6,26].

Finally, the concept of “mass walking” warrants specific consideration in this context. Unlike conventional clinical or laboratory gait assessments, pilgrimage walking involves prolonged repetitive ambulation under crowded and often stressful conditions, where motivation, religious commitment, and social context may influence performance. It is possible that the motivational factors inherent to the pilgrimage experience partially offset the physiological limitations during short-duration assessments. Future studies should therefore incorporate psychological or motivational measures to better disentangle physiological capacity from motivational drive and comprehensively model fall risk during sustained mass walking.

## 5. Limitations

This study acknowledges several limitations that warrant consideration. The employment of convenience sampling and clinic-based recruitment may restrict the generalizability of the findings to pilgrims who do not participate in pre-Hajj screening. The cross-sectional design precludes causal inference and does not account for the actual incidence of falls during pilgrimage activities. Additionally, the absence of systematically collected data on medication use and neurological conditions, due to the pragmatic field-based nature of the screening setting, represents unmeasured variables that may have influenced gait performance and functional mobility outcomes. These factors constitute potential sources of residual confounding that could impact fall-risk classification. Fatigue, despite its relevance to prolonged walking, was neither directly manipulated nor longitudinally measured. Furthermore, motivational factors inherent to the religious context may have positively influenced performance. Future prospective studies incorporating longitudinal follow-up and psychological measures are warranted.

## 6. Conclusions

This study establishes the feasibility and utility of an integrated functional mobility and spatiotemporal gait screening framework for differentiating fall risk among pilgrims. By translating objective measures into an operational, color-coded classification system, this approach offers a scalable, field-feasible solution for proactive mobility management and fall-risk mitigation in mass-gathering environments. Future longitudinal validation is crucial to confirm its long-term effectiveness and impact on pilgrim safety.

## Figures and Tables

**Table 1:** Physical and Clinical Characteristics of Participants According to Fall-Risk Group.

Characteristic	Low Fall Risk *n* = 103	Moderate Fall Risk *n* = 24	High Fall Risk *n* = 29
Age group			
<50 years	43 (41.7%)	5 (20.8%)	2 (6.9%)
≥50 years	60 (58.3%)	19 (79.2%)	27 (93.1%)
Sex			
Male	61 (59.2%)	8 (33.3%)	7 (24.1%)
Female	42 (40.8%)	16 (66.7%)	22 (75.9%)
Chronic disease			
Yes	39 (37.9%)	12 (50.0%)	21 (72.4%)
No	64 (62.1%)	12 (50.0%)	8 (27.6%)
Musculoskeletal disorder			
Yes	46 (44.7%)	20 (83.3%)	23 (79.3%)
No	57 (55.3%)	4 (16.7%)	6 (20.7%)
Need cane			
Yes	0 (0%)	22 (91.7%)	29 (100.0%)
No	103 (100%)	2 (8.3%)	0 (0%)
Card colour distribution			
Green	0 (0%)	24 (100%)	0 (0%)
Yellow	0 (0%)	0 (0%)	29 (100%)
No need	103 (100%)	0 (0%)	0 (0%)

*n*: number of participants in each fall-risk group.

**Table 2:** Characteristics of Spatiotemporal Gait, Functional Mobility, and Demographics According to Fall-Risk Classification.

Variable	Low Fall Risk Median (Q1–Q3)	Moderate Fall Risk Median (Q1–Q3)	High Fall Risk Median (Q1–Q3)	H (df = 2)	*p*-Value	ε^2^ (Effect Size)
Age (years)	52.00 (41.00–61.25)	56.00 (50.00–61.00)	60.00 (56.50–65.25)	19.97	<0.001	0.10
Body Mass Index (kg/m^2^)	27.49 (24.24–32.19)	30.30 (26.16–38.65)	30.92 (24.42–33.59)	2.20	0.332	0.00
Cadence (steps/min)	82.50 (73.00–91.75)	75.00 (66.25–82.00)	72.00 (55.00–81.00)	14.77	0.001	0.07
Average Half-Step Length (cm)	44.00 (39.00–50.00)	36.50 (33.75–43.25)	35.00 (31.50–37.00)	37.67	<0.001	0.20
Walking Speed (cm/s)	37.46 (28.23–44.05)	27.88 (22.48–32.02)	23.04 (18.87–30.45)	27.14	<0.001	0.14
Test Duration (s)	9.00 (7.00–12.00)	10.50 (8.75–14.00)	15.00 (11.00–29.00)	11.54	0.003	0.05
Symmetry Index (%)	9.96 (4.49–19.15)	10.17 (4.98–19.45)	15.47 (10.29–21.10)	0.28	0.871	0.00
TUG—Before (s)	9.00 (7.00–10.00)	13.00 (10.75–14.25)	15.00 (10.00–18.00)	110.58	<0.001	0.62

Values are presented as median (Q1–Q3) represents the interquartile range (25th–75th percentile), Group comparisons were performed using the Kruskal–Wallis H test, Effect size is reported as epsilon-squared (ε^2^), TUG: Timed Up and Go test.

**Table 3:** Association Between TUG Performance and Demographic, Anthropometric, Gait, and Functional Mobility Variables.

Variable	Spearman ρ	*p*-Value
Age (years)	0.134	0.107
Sex (M/F)	−0.357	<0.001
Body Mass Index (kg/m^2^)	0.246	0.003
Weight-bearing time (right) (s)	0.584	<0.001
Weight-bearing time (left) (s)	0.461	<0.001
Test duration (s)	0.376	<0.001
Number of steps acquired	0.356	<0.001
Symmetry Index (%)	0.058	0.499
Cadence (steps/min)	−0.420	<0.001
Walking speed (cm/s)	−0.577	<0.001
Half-step length (cm)	−0.617	<0.001

Spearman’s rank correlation coefficient (ρ) was used to assess associations between fall-risk classification, *p*-value < 0.05 was considered statistically significant. Positive ρ values indicated correlation, while negative values indicate an inverse correlation. Sex was coded as male = 1 and female = 0.

## Data Availability

Data for the current study will be available upon reasonable request from the corresponding author.

## References

[1] Memish Z.A., Steffen R., White P., Dar O., Azhar E.I., Sharma A., Zumla A. (2019). Mass gatherings medicine: Public health issues arising from mass gathering religious and sporting events. Lancet.

[2] Steffen R., Bouchama A., Johansson A., Dvorak J., Isla N., Smallwood C., Memish Z.A. (2012). Non-communicable health risks during mass gatherings. Lancet Infect. Dis..

[3] Al-Jazzar W., Almudaimegh K., Nooh R., Choudhry A., Al-Rabeah A. (2002). Pattern of injuries presenting at health facilities in Makkah and Mina during Hajj 1422 H. Saudi Epidemiol Bull..

[4] Alghamdi G.A., Alghamdi F.A., Almatrafi R.M., Sadis A.Y., Shabkuny R.A., Alzahrani S.A., Alessa M.Q., Hafiz W.A. (2024). The Prevalence of Musculoskeletal Injuries Among Pilgrims During the 2023 Hajj Season: A Cross-Sectional Study. Cureus.

[5] Ahmad H., Alshehri R., Ghazwani M., Alqthmi A., Hamzah A., Albeshri T., Khiyami A.J., Attiah M. (2025). Prevalence of musculoskeletal injuries in Hajj 2024. Int. J. Med. Dev. Ctries..

[6] World Health Organization (2015). Public Health for Mass Gatherings: Key Considerations.

[7] Koski A., Kouvonen A., Sumanen H. (2020). Preparedness for Mass Gatherings: Factors to Consider According to the Rescue Authorities. Int. J. Environ. Res. Public Health.

[8] van Exter S.H., Koenders N., van der Wees P., van den Berg M.G.A. (2024). A systematic review of the psychometric properties of physical performance tests for sarcopenia in community-dwelling older adults. Age Ageing.

[9] Podsiadlo D., Richardson S. (1991). The Timed “Up & Go”: A Test of Basic Functional Mobility for Frail Elderly Persons. J. Am. Geriatr. Soc..

[10] Cho B., Scarpace D., Alexander N.B. (2004). Tests of Stepping as Indicators of Mobility, Balance, and Fall Risk in Balance-Impaired Older Adults. J. Am. Geriatr. Soc..

[11] Olivares P.R., Gusi N., Prieto J., Hernandez-Mocholi M.A. (2011). Fitness and health-related quality of life dimensions in community-dwelling middle aged and older adults. Health Qual. Life Outcomes.

[12] Shumway-Cook A., Brauer S., Woollacott M. (2000). Predicting the Probability for Falls in Community-Dwelling Older Adults Using the Timed Up & Go Test. Phys. Ther..

[13] Beauchet O., Fantino B., Allali G., Muir S.W., Montero-Odasso M., Annweiler C. (2011). Timed up and go test and risk of falls in older adults: A systematic review. J. Nutr. Health Aging.

[14] Hausdorff J.M. (2005). Gait variability: Methods, modeling and meaning. J. Neuroeng. Rehabil..

[15] Maki B.E. (1997). Gait Changes in Older Adults: Predictors of Falls or Indicators of Fear?. J. Am. Geriatr. Soc..

[16] Verghese J., Holtzer R., Lipton R.B., Wang C. (2009). Quantitative Gait Markers and Incident Fall Risk in Older Adults. J. Gerontol. Ser. A Biomed. Sci. Med. Sci..

[17] Granacher U., Wolf I., Wehrle A., Bridenbaugh S., Kressig R.W. (2010). Effects of muscle fatigue on gait characteristics under single and dual-task conditions in young and older adults. J. Neuroeng. Rehabil..

[18] Mickle K.J., Munro B.J., Lord S.R., Menz H.B., Steele J.R. (2010). Foot Pain, Plantar Pressures, and Falls in Older People: A Prospective Study. J. Am. Geriatr. Soc..

[19] Mickle K.J., Munro B.J., Lord S.R., Menz H.B., Steele J.R. (2011). Gait, balance and plantar pressures in older people with toe deformities. Gait Posture.

[20] Lord S.R., Menz H.B., Tiedemann A. (2003). A Physiological Profile Approach to Falls Risk Assessment and Prevention. Phys. Ther..

[21] Bateni H., Maki B.E. (2005). Assistive devices for balance and mobility: Benefits, demands, and adverse consequences. Arch. Phys. Med. Rehabil..

[22] Willson J., Torry M.R., Decker M.J., Kernozek T., Steadman J.R. (2001). Effects of walking poles on lower extremity gait mechanics. Med. Sci. Sports Exerc..

[23] Passfield J. S-MT02: Prescribe, Train and Review of Walking Aids [Internet]. https://www.health.qld.gov.au/__data/assets/pdf_file/0028/675118/s-mt02.pdf.

[24] Bazyar J., Farrokhi M., Salari A., Khankeh H.R. (2020). The Principles of Triage in Emergencies and Disasters: A Systematic Review. Prehospital Disaster Med..

[25] Christian M.D. (2019). Triage. Crit. Care Clin..

[26] Ranse J., Hutton A., Keene T., Lenson S., Luther M., Bost N., Johnston A.N.B., Crilly J., Cannon M., Jones N. (2017). Health Service Impact from Mass Gatherings: A Systematic Literature Review. Prehospital Disaster Med..

[27] Arbon P. (2007). Mass-Gathering Medicine: A Review of the Evidence and Future Directions for Research. Prehospital Disaster Med..

[28] Atkinson G., Nevill A.M. (1998). Statistical Methods For Assessing Measurement Error (Reliability) in Variables Relevant to Sports Medicine. Sports Med..

[29] Bohannon R.W. (2006). Reference Values for the Timed Up and Go Test. J. Geriatr. Phys. Ther..

[30] Hills A.P., Hennig E.M., Byrne N.M., Steele J.R. (2002). The biomechanics of adiposity—Structural and functional limitations of obesity and implications for movement. Obes. Rev..

[31] freeMed-User Manual rev 2.0 USER MANUAL [Internet]. http://www.sensormedica.com.

[32] Giacomozzi C. (2010). Appropriateness of plantar pressure measurement devices: A comparative technical assessment. Gait Posture.

[33] Field A. (2014). Discovering Statistics Using IBM SPSS Statistics.

